# *apterous* Brain Neurons Control Receptivity to Male Courtship in *Drosophila Melanogaster* Females

**DOI:** 10.1038/srep46242

**Published:** 2017-04-12

**Authors:** Márcia M. Aranha, Dennis Herrmann, Hugo Cachitas, Ricardo M. Neto-Silva, Sophie Dias, Maria Luísa Vasconcelos

**Affiliations:** 1 Champalimaud Neuroscience Programme, Champalimaud Centre for the Unknown, 1400-038 Lisbon, Portugal; 2 Instituto Gulbenkian de Ciência, 2780-156 Oeiras, Portugal

## Abstract

Courtship behaviours allow animals to interact and display their qualities before committing to reproduction. In fly courtship, the female decides whether or not to mate and is thought to display receptivity by slowing down to accept the male. Very little is known on the neuronal brain circuitry controlling female receptivity. Here we use genetic manipulation and behavioural studies to identify a novel set of neurons in the brain that controls sexual receptivity in the female without triggering the postmating response. We show that these neurons, defined by the expression of the transcription factor *apterous,* affect the modulation of female walking speed during courtship. Interestingly, we found that the *apterous* neurons required for female receptivity are neither *doublesex* nor *fruitless* positive suggesting that *apterous* neurons are not specified by the sex-determination cascade. Overall, these findings identify a neuronal substrate underlying female response to courtship and highlight the central role of walking speed in the receptivity behaviour.

During courtship, typically the male does the displays and the female decides whether or not to mate^1^. The female’s decision of accepting or not a courting male is an important component of sexual selection and therefore plays a key role in the evolution of a given species. Nevertheless very little is known about how the female brain processes internal and external cues to generate a response in this behavioural context.

In fruit flies, courtship represents a complex set of behaviours^2^,^3^. The male orients towards the female, follows her, extends the wing and vibrates it to generate the courtship song. Then, the male taps and licks the female and attempts copulation^2^,^3^. If the female is receptive, they copulate. The behaviour exhibited by the female is less evident. When courted, a virgin female may walk away until she eventually stops and accepts the male^4^,^5^. She also displays a few behavioural elements that may be interpreted as mild rejection responses such as wing fluttering, ovipositor extrusion, decamping, fending, kicking, abdominal preening and droplet emission^4^,^6^,^7^,^8^. These behavioural responses are exhibited by both receptive and unreceptive flies, but at different frequencies^4^,^9^. It has been suggested that abdominal preening and the ovipositor extrusion are predictors of the female willingness to copulate^9^. However, the frequency of each of these elements is very low and the display is quite subtle. A decrease in female’s walking speed may be the most reliable readout associated with virgin female receptivity^4^,^5^,^10^,^11^.

The behaviour of the female changes dramatically once she has copulated^12^,^13^. She becomes temporarily unreceptive, lays an order of magnitude more eggs and even changes her nutrient preference^12^,^13^,^14^,^15^. This change in behaviour is called postmating switch and is triggered by the sex-peptide, which is transferred from the male to the female during copulation^16^,^17^,^18^. In the past years, a specific set of sensory and second order neurons carrying the sex-peptide information from the reproductive system to the dorsal protocerebrum have been identified^19^,^20^,^21^,^22^. Though the connectivity is less clear, two other sets of neurons in the abdominal ganglion contribute to the postmating responses^23^,^24^.

Female receptivity is thought to be dependent on the assessment of the male’s quality. In this respect, the detection of the male-specific pheromone cisVaccenyl acetate (cVA) and the male courtship song are particularly relevant for the female to evaluate the courting male. The courtship song is perceived in a subset of mechanosensory neurons of the auditory system that converge onto the antennal mechanosensory and motor centre (AMMC). More recently, a specific set of second order neurons dedicated to sensing the courtship song have been identified^25^,^26^,^27^. In the olfactory system cVA is detected through sensory neurons that express the odorant receptor Or67d^28^. cVA information is then transferred to the lateral horn where it is processed by sexually dimorphic neurons that express the transcription factor *fruitless*^29^,^30^,^31^. More recently, *spinster* mutant mosaics revealed that loss of *spinster* function in VA1v olfactory projection neurons also leads to receptivity reduction^32^.

Classic mosaic studies identified a dorsal region of the brain that needs to be genetically female for the animal to be receptive^33^. In a different region of the brain, two *doublesex-*expressing clusters of neurons, pC1 and pCd, were shown to be required for female receptivity^34^. pCd neurons receive cVA information and pC1 cluster respond to both cVA and the courtship song, suggesting that it could act as an integration centre of multiple courtship stimuli. The abdominal ganglion of the ventral nerve chord (VNC) was also recently shown to be involved in virgin female (i.e., premating) behaviours^10^. There, a cluster of *Abdominal*-*B*-expressing neurons controls pausing, which was identified as a discrete component of the female behaviour that signal a receptive state^5^,^10^. Though some elements of the circuit for female receptivity have been identified, the circuit is not well understood.

Here we show that activity in neurons that are defined by the expression of *apterous* is required for female receptivity. The behavioural alterations associated with manipulation of *apterous* neurons are distinct from those observed in postmating responses, suggesting that they may be a component of virgin female receptivity circuit. We found that *apterous* neurons in the brain are required for the appropriate reduction of the walking speed in the presence of a courting male but not for the recognition of the presence of the male. We show that the lack of reduction in walking speed is a consequence rather then a cause of low receptivity. The *apterous* neurons in the brain involved in the phenotype are neither *doublesex* nor *fruitless* positive indicating that they are not part of a sexually differentiated circuit.

## Results

### Silencing *apterous* neurons impairs mating

**Figure 1:**
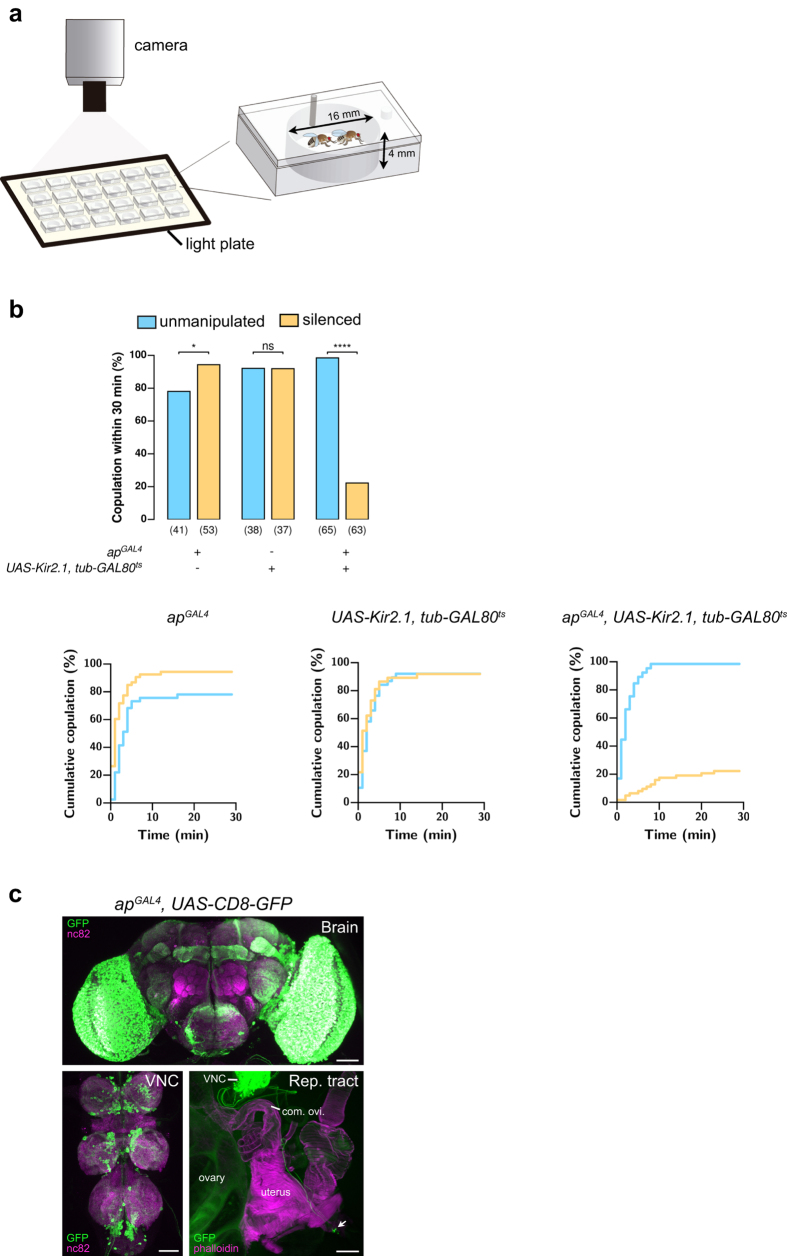
Silencing *apterous* neurons in females impairs mating. (**a**) Schematic representation of the behavioural setup to test female receptivity. Mating arena containing mating pairs is highlighted. (**b**) Mating of virgin females. Genotypes indicate virgin females. n values shown in parentheses. n.s., not significant, ****p < *0.0001*, Fisher’s exact test. (**c**) Expression pattern of *ap*^*GAL4*^ in the CNS and reproductive system of adult females. Flies were aged for 8 to 9 days (CNS) and 4 days (reproductive system) at room temperature prior to dissection. GAL4-driven expression is shown in green while the synaptic marker nc82 or Alexa 594-conjugated phalloidin is shown in magenta. Arrow indicates the cell bodies found in the reproductive system. Scale bar represents 50 μm for the Brain and VNC and 100 μm for the reproductive system.

Female flies mutant for the *apterous* gene have low sexual receptivity^35^. Apterous is a LIM-homedomain transcription factor with a role in tissue specification, pattern formation and axon guidance^36^,^37^,^38^. As a first approach to identify neurons involved in female receptivity behaviour, we wondered if *apterous*-positive neurons could play a role in female sexual behaviours. For this, we used an *apterous* enhancer trap line, *ap*^*md544*^ (hereafter referred to as *ap*^*GAL4*^), which was previously shown to accurately report expression of the *apterous* gene^39^. We silenced these neurons by expressing the inwardly rectifier potassium channel, *Kir2.1*^40^, that hyperpolarizes the neurons and therefore decreases the probability of firing an action potential. In order to prevent developmental defects that could arise from a permanent silencing, we restricted Kir2.1 expression to adult stage by using a temperature sensitive *GAL80 (GAL80*^*ts*^), that blocks *Kir2.1* expression at lower temperature, 18 °C, but allows expression at higher temperatures (above 29 °C)^41^. Mating was evaluated at 25 °C by pairing single wild-type males and temperature treated females in a plexiglass chamber (Fig. 1a). We found a drastic reduction in the number of flies that copulated after silencing *ap*^*GAL4*^ neurons (Fig. 1b). To confirm that the copulation phenotype results from disruption of neuronal function, we used *elav-GAL80*^20^ to block *Kir2.1* expression specifically in neurons (Figure S1a). In this manipulation the pairs copulate, suggesting that indeed the phenotype results from neuronal silencing.

*apterous* expression is widespread in the nervous system of the adult female (Fig. 1c). However, there is no expression in neurons that, at the sensory level, are known to be important for female receptivity. More specifically, there is no innervation of the antennal lobe and very faint innervation of the antennal mechanosensory and motor centre (AMMC) meaning that first and second order neurons of the olfactory and auditory systems are unlikely to contribute to the phenotype (higher magnification image in Figure S1b). In the VNC, clusters of neurons are detected along the midline of each ganglia, as well as in the abdominal ganglion, the anatomical foci for copulatory behaviours in both sexes^42^. Inspection of the uterus reveals the presence of four to six cell bodies near the ovipositor (n = 4), likely sending projections to the VNC. Notably, no neurons were detected at the location of the sex-peptide sensory neurons^20^,^21^. In contrast, the optic lobes are widely labelled but a role for visual information in the female in this behavioural context has not been reported. To investigate whether the inability to process visual stimuli could explain the reduced mating, we tested the *no receptor potential A (norpA*) mutant flies, that have no phototransduction^43^. We observed that the mating of these flies is not changed (Figure S1c). The mushroom body (MB) is thought to not play a role in this behavioural paradigm. To confirm this, we expressed *Kir2.1* in the MB using the *OK107-GAL4* driver line and observed a wild-type phenotype (Figure S1d). Together, these results suggest that a subset of *apterous* neurons that do not include those comprised in the visual system and the MB are necessary for normal mating.

### Silencing *apterous* neurons in the brain impairs mating

**Figure 2:**
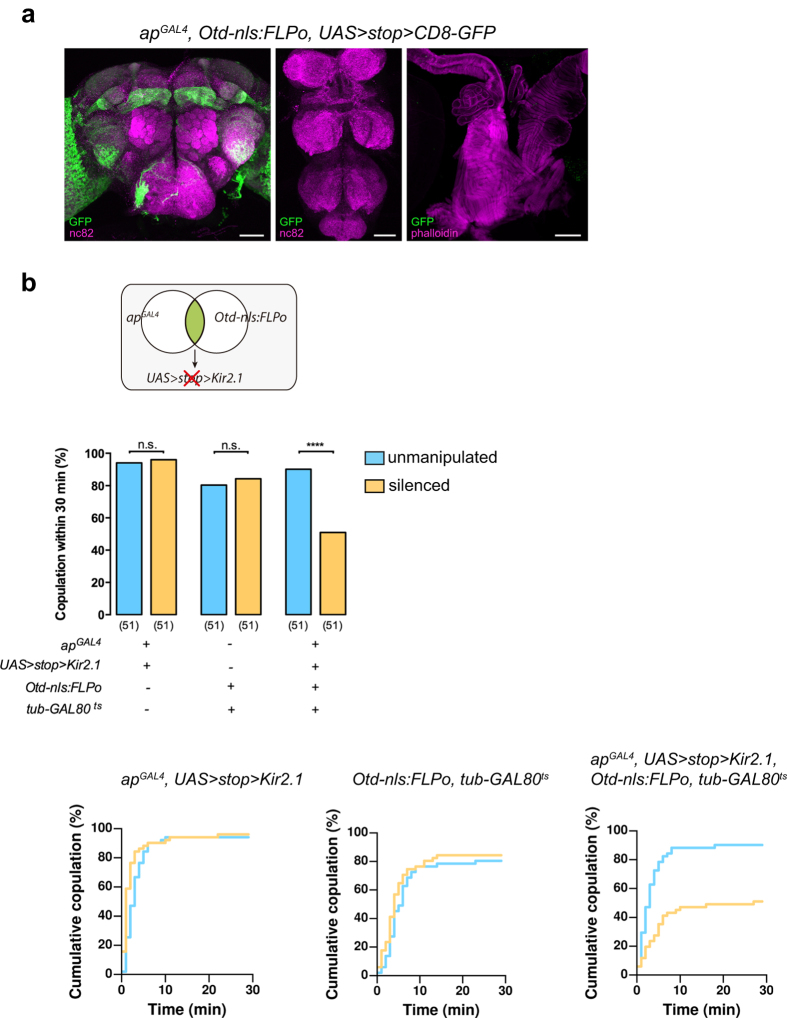
*apterous* neurons located in the brain are responsible for the mating phenotype. (**a**) *ap*^*GAL4*^*/Otd-nls:FLPo* intersecting neurons labelled in 3 to 4 day old females. The reproductive system was dissected from females that also carried a *tub-GAL80*^*ts*^ transgene; flies were incubated at 30 °C for 24 h prior to dissection. Intersecting neurons were visualized with anti-GFP (green) and the tissue counterstained with nc82 or phalloidin (magenta). Scale bar represents 50 μm for the Brain and VNC and 100 μm for the reproductive system. (**b**) Schematic depicting the intersectional strategy employed and quantification of mating of virgin females with silenced central brain *apterous* neurons. Genotypes shown correspond to those of virgin females. n values shown in parentheses. n.s., not significant, ****p < *0.0001*, Fisher’s exact test.

We next asked which are the *apterous* neurons responsible for the mating defect and set out to determine whether they are located in the central brain or the remainder of the nervous system. We used an intersectional approach to selectively express *Kir2.1* in *apterous* positive brain neurons. To this end, we used an *UAS* > *stop* > *Kir2.1* transgene in combination with *Otd-nls:FLPo* that expresses flipase (*FLP*) specifically in the brain^44^, with the exception of the gnathal ganglia (Fig. 2a). We observed that females with *apterous* brain neurons silenced copulate less than controls (Fig. 2b). This indicates that the *apterous* neurons that contribute to the mating phenotype are located in the brain. It is important to notice that the observed effect on copulation is not quite as strong when compared to silencing of all *apterous* neurons. The difference could be explained by a small contribution of *apterous* neurons outside of the brain to the phenotype, or that the flipase-mediated recombination did not take place in all the neurons of the intersection.

To exclude a possible contribution coming from *apterous* neurons outside of the brain we set out to do a complementary set of experiments. In these experiments, we inactivate the *apterous* neurons that are not located in the central brain using *Otd-nls:FLPo* combined with *tub* > *stop* > *GAL80*^45^ and *UAS-Kir2.1*. In this combination, expression of *FLP* will result in *GAL80* expression in the central brain where it will prevent Kir2.1 expression (Figure S2a, *apterous*-positive neurons outside of the brain visualized with GFP expression). We observed that upon silencing of the *apterous* neurons outside of the brain, the flies remain receptive (Figure S2b), suggesting that activity of *apterous* neurons that are located in the gnathal ganglion and in the VNC is not essential for female mating.

In an attempt to restrict the candidate neurons, we silenced the neurons at the intersection of *apterous* and glutamatergic or GABAergic or cholinergic neurons (Figure S3). Silencing the neurons of these intersections did not result in a reduction in mating.

### Silencing of *apterous* neurons in the brain does not lead to immature virgin or postmating behavioural responses

**Figure 3:**
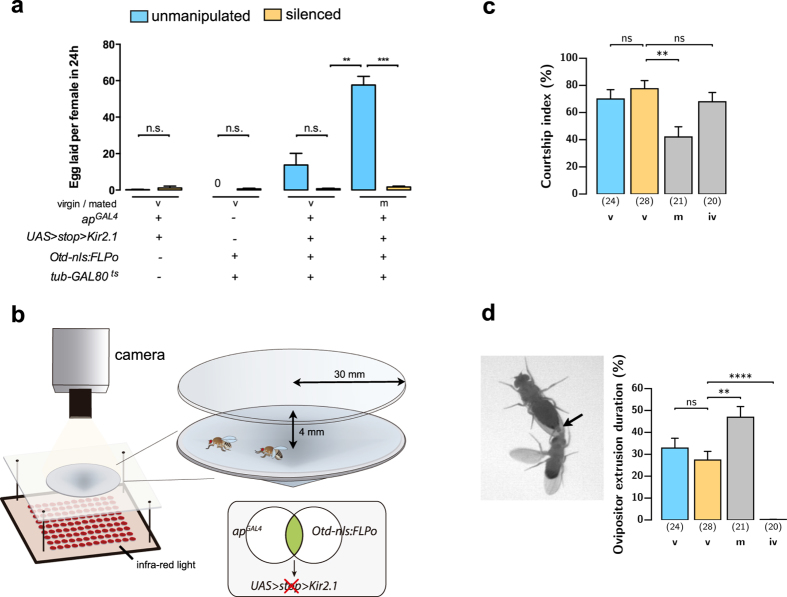
Females with silenced *apterous* brain neurons do not exhibit hallmarks of the postmating switch or immature virgin behaviour. (**a**) Egg laying of virgin and mated flies. Genotypes indicate females. n.s., not significant, **p < *0.01*, Mann Whitney test and ***p < *0.001*, unpaired t test with Welch’s correction, mean ± SEM, n = 5–8. (**b**) Schematic representation of a different behavioural setup developed to quantify and characterize receptivity behaviour. A conical-shaped arena containing mating pairs is highlighted. All behavioural analysis were performed in flies with *ap*^*GAL4*^*/Otd-nls:FLPo* intersecting neurons silenced, as depicted in the scheme. See also Figure S4. (**c**) Male courtship index towards females of the indicated mating status, during the first 10 min of courtship or until copulation. v-virgin; m-mated; iv-immature virgin. n values shown in parentheses. n.s., not significant, **p < *0.01*, Mann Whitney test, mean ± SD. (**d**) Ovipositor extrusion duration within courtship period of females with the indicated mating status. A video frame showing the relevant behaviour is shown. Arrow indicates the extruded ovipositor. v-virgin; m-mated; iv-immature virgin. n values shown in parentheses. n.s., not significant, **p < 0.01, ****p < *0.0001*, Mann Whitney test, mean ± SD.

In flies, as in many other insect species, mating leads to an increase in egg laying^13^. Inhibition of the sex peptide sensory neurons and the sex peptide abdominal ganglion (SAG) neurons in virgin females results in a mated-like behaviour: they become unreceptive and increase their egg laying^20^,^21^,^22^. To test whether silencing *apterous* neurons leads to postmating responses we measured egg laying of virgin flies after silencing *apterous* brain neurons (which we will call henceforth *apterous*-silenced virgin females). The *apterous*-silenced virgin females did not show an increase in egg laying (Fig. 3a). If at all they laid even fewer eggs than control virgin females. We repeated the same experiment with mated flies. We allowed the flies to mate before the temperature shift and tested egg laying after neuronal silencing. Here we see a dramatic decrease in the number of eggs laid.

Mated flies elicit less courtship from the male^46^ and display high levels of rejection behaviours such as ovipositor extrusion^4^. We set out to measure these parameters not only in mated and virgin *apterous*-silenced flies but also in immature virgins that are unreceptive^12^. To better track the interactions of the male and female fly, we repeated the receptivity experiments in larger arenas that are also sloped^47^, i.e., without lateral walls so that the camera captures either the ventral or the dorsal side of the flies but not the flanks (Fig. 3b). Here we recorded the pairs for 20 min instead of 30 min because most matings occur within 20 min (Fig. 2b). We observed the same reduction in receptivity of *apterous*-silenced virgin females in the new setup (Figure S4a).

We observed that *apterous*-silenced flies are courted at high levels, comparable to virgin control flies and more intensively than mated flies (Fig. 3c). Immature virgin females also elicit courtship even though they do not copulate. We also measured the time until the male begins courtship (latency to court), wing extension (as a proxy for courtship song), the distance between the flies during courtship and orientation of the male relative to the female. All are comparable between videos with *apterous*-silenced females and control females (Figure S5). Therefore, male courtship activity does not appear to be the source of reduced copulation rate but instead it results from reduced female receptivity. Regarding rejection responses, we found that *apterous*-silenced females have an ovipositor extrusion pattern similar to virgin control females (Fig. 3d). Immature virgins, on the other hand, do not extrude the ovipositor. This observation is in agreement with what was previously observed^10^ and sets the *apterous*-silenced female phenotype apart from immature virgin behaviour.

The quantifications of egg laying, elicited courtship and ovipositor extrusion all indicate that the reduction in receptivity observed upon silencing of *apterous* neurons is separable from hallmarks of the unreceptive periods when the female is immature and after mating.

### *apterous*-silenced females exhibit higher walking speed in the presence of a courting male

**Figure 4:**
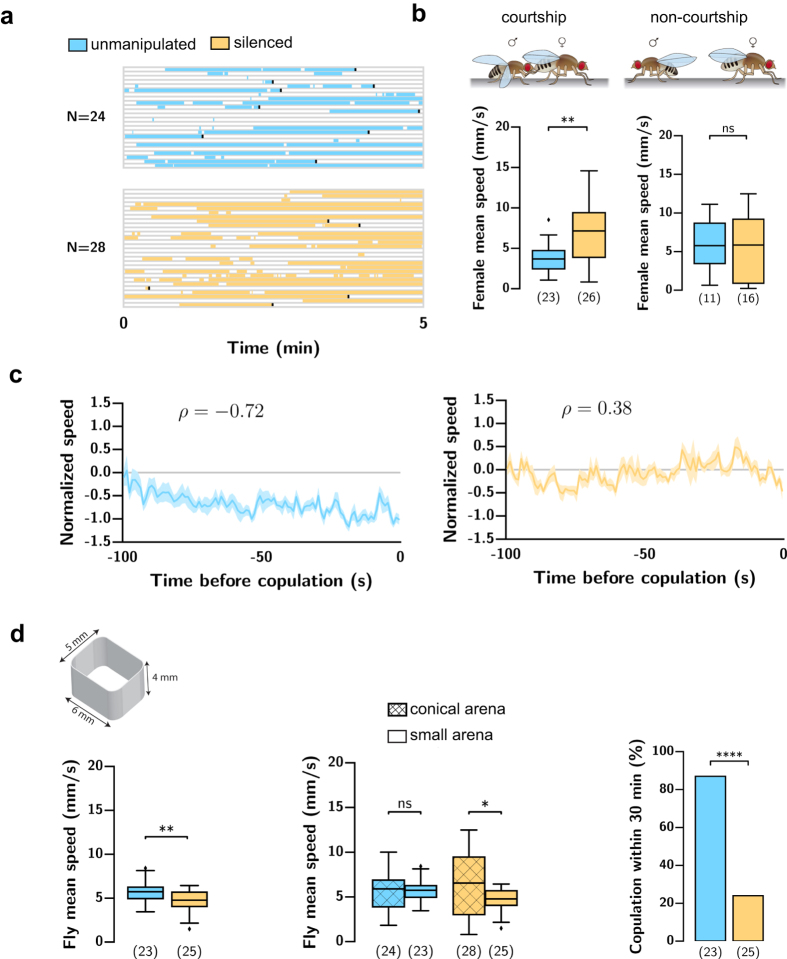
Silencing *apterous* brain neurons affects the modulation of female walking speed during courtship likely as a result of reduced receptivity. (**a**) Courtship bouts in the first five minutes of video of virgin control and virgin with silenced brain *apterous* neurons. Each line is one video. The black rectangle indicates copulation. (**b**) Female velocity during courtship periods (‘courtship’) and outside of courtship periods (‘non-courtship’). During courtship: *p < *0.05*, Mann-Whitney U test. During non-courtship: n.s., not significant, unpaired t test. (**c**) Normalized changes in female velocity before copulation. To quantify the association between velocity and time to copulation we calculated rank correlations. Spearman’s rank correlation: Control females, 20 videos, 30 frames bin, *p* < *0.0001*; *apterous*-silenced females that copulated, 15 videos, 30 frame bin, *p* < *0.0001*. (**d**) Schematic of the small arena where the experiments were performed. Fly velocity and quantification of mating. n values shown in parentheses. n.s., not significant, *p < 0.05, **p < 0.01, ****p < *0.0001* unpaired t test for the fly speed in the small arena, Mann-Whitney U test for speed comparison across arenas and Fisher’s exact test for mating.

We next looked at the locomotor activity displayed in the context of courtship behaviours. Fly courtship occurs in bouts (Fig. 4a). We compared the velocity of *apterous*-silenced females and control virgins during the periods of courtship and non-courtship and found that *apterous*-silenced females walk faster than the control virgins during courtship bouts but have the same walking speed in the periods when the male is not courting (Fig. 4b). To test whether the higher speed is a specific response to a courting male, we repeated the experiment this time pairing the test female with a wild-type virgin female (Figure S6a). We labelled as courtship-like the moments in the video where the two females were below 5.5 mm apart because in male-female videos this corresponds to 95% likelihood of courtship (see methods). During courtship-like periods, the mean velocity of *apterous*-silenced females did not differ from the mean velocity of virgin control females without neuronal silencing, in contrast with what we observe in male-female videos. This suggests that the higher speed of *apterous*-silenced females compared to control females is specifically displayed in the presence of a courting male.

The increased average speed could be the result of decreased pausing^10^. We measured pausing during courtship and found no difference with control females (Figure S6b).

To further dissect the velocity phenotype, we examined the progression of the velocity of the females in 100 s leading to copulation^11^. There is a steady trend in reduction of speed for control females until copulation (Fig. 4c, rank correlation ρ = −0.72, see also Figure S7). *apterous*-silenced females that copulated, however, do not show the progressive reduction in velocity (rank correlation ρ = 0.38).

Are *apterous*-silenced females not copulating because they cannot reduce their speed or do they not reduce their speed because they are not receptive? To address this question we performed mating experiments in arenas where flies do not have room to walk away. The arenas are 4 × 5 × 6 mm (Fig. 4d). In this setup we cannot track the velocity of the male and the female separately. Instead, we measure the speed of any fly because, when measured in the conical arenas, male and female velocities are similar (Figure S8). In the small arenas, *apterous*-silenced females do not walk faster than control females. In fact, they appear to walk even slower (Fig. 4d). We also compared the mean speed of *apterous*-silenced females in the conical arena and the small arena to verify that indeed the speed is lower in the small arena. In this context *apterous*-silenced females still show a severe reduction in mating within 30 min.

In sum, *apterous*-silenced females fail to reduce their velocity in response to male courtship because they are not receptive.

### *apterous* neurons are not specified by the sex-determination cascade

**Figure 5:**
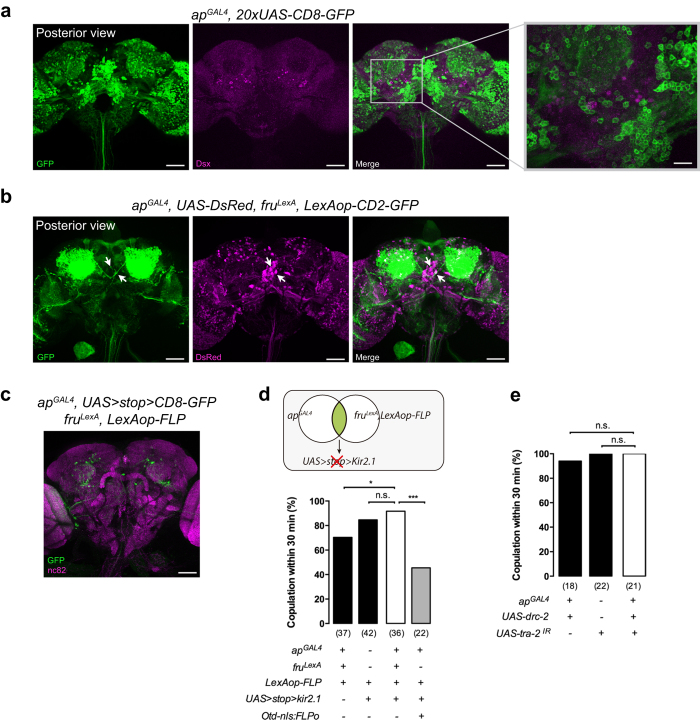
*apterous* neurons required for female receptivity are not part of the *doublesex* and *fruitless* circuitry. (**a**) *ap*^*GAL4*^/UAS-CD8-GFP female brain (3 days) stained with anti-GFP (green) and anti-Dsx (magenta). No coexpression between *ap*^*GAL4*^ and Dsx was detected in the brain. Scale bar represents 50 μm except inset where it represents 15 μm. (**b**) Labeling of *apterous* and *fruitless*-positive neurons in the female brain (3–6 days) using *ap*^*GAL4*^*/UAS-DsRed* (magenta) and *fru*^*LexA*^*/LexAop-CD2-GFP* (green). Coexpression of *ap*^*GAL4*^ and *fru*^*LexA*^ was detected in two neurons in the posterior brain (arrows). Scale bar represents 50 μm. (**c**) *ap*^*GAL4*^*/fru*^*LexA*^ intersecting neurons labelled in females aged for 15 to18 days at room temperature. Intersecting neurons were visualized with anti-GFP (green) and nc82 (magenta). Scale bar represents 50 μm. (**d**) Quantification of mating of virgin females in which overlapping *apterous* and *fruitless* circuitry was silenced with a schematic representation of the manipulation employed depicted. Genotypes indicate virgin females. n values shown in parentheses. n.s., not significant, *p < 0.05, ***p < *0.001*, Fisher’s exact test. (**e**) Quantification of mating of virgin females with *ap*^*GAL4*^ neurons ‘masculinized’. Genotypes indicate virgin females. n values shown in parentheses. n.s., not significant, Fisher’s exact test.

The transcription factors *fruitless (fru*) and *doublesex (dsx*) establish the neuronal circuits of sexually dimorphic behaviours^6^. We asked whether *apterous* neurons in the central brain are *fruitless* or *doublesex* positive. We performed Doublesex immunostaining in female adult brains where GFP expression is driven by *ap*^*GAL4*^ (Fig. 5a). Surprisingly, we observed no colocalization between *apterous* and Doublesex markers (n = 3). We also compared expression of *ap*^*GAL4*^ and Doublesex in female pupal brains but no colocalization was observed (data not shown). Sex-specific Fruitless protein is only produced in males (Fru^M^) as a consequence of sex-specific splicing^48^,^49^ Females do not produce Fru^M^ but the P1 promoter involved in the expression of the sex-specific *fru* transcript, is transcriptionally active in females^50^,^51^. *fru*^*GAL4*^ and *fru*^*LexA*^ driver lines allow access to these cells^50^,^51^,^52^. We next checked a possible overlap between *fru*^*LexA*^ and *ap*^*GAL4*^ neurons (Fig. 5b). We saw overlap in two posterior medial cell bodies and in a small subset of Kenyon cells (n = 3). To test the role of the overlapping set of neurons in female receptivity, we used an intersectional strategy that allows *Kir2.1* expression only in the neurons that coexpress *fru*^*LexA*^ and *ap*^*GAL4*^ (Fig. 5c and d). This manipulation has no effect on mating, indicating that the receptivity phenotype we observe when silencing *apterous* neurons is not part of the circuitry in which *fru* P1 promoter is expressed.

We further investigated the possibility of the sexual differentiation cascade specifying the *apterous* neurons. The splicing factors *transformer (tra*) and *transformer-2 (tra2*) work in conjunction to control female specific splicing of *fruitless* and *doublesex*^53^. Females carrying a mutation in the *tra-2* gene exhibit male-like morphology and behaviour^54^,^55^,^56^. We used RNA interference to remove endogenous Tra-2 specifically from the *apterous* neurons in the female (Fig. 5e). In other words, we masculinized *apterous* neurons in the female. The treated flies did not show a change in mating suggesting that the *apterous* neurons do not need to be genetically female to allow the proper display of female specific behaviour. Importantly, we verified that the *UAS-tra-2-IR* transgene was indeed masculinizing cells by expressing it ubiquitously with *actin5C-GAL4* and observed that females looked like males (data not shown).

Altogether these experiments indicate that *apterous* neurons are not directly part of a sexually differentiated circuit.

## Discussion

Reproductive behaviours are essential for the survival and fitness of the species. In *Drosophila melanogaster*, as in many other species, the decision of whether or not to mate is made by the female. However, we still have a very poor understanding about the behaviour displayed by the virgin female fly and the neuronal circuits underlying it. We set out to identify neurons involved in the response of virgin females to courting males. We show that female virgin flies with compromised activity in *apterous* neurons in the brain display a substantial reduction in copulation. What is specifically changed in the premating behaviour of *apterous*-silenced females? *apterous*-silenced females do not slowdown during courtship even though they do recognize they are in the presence of a courting male because they extrude the ovipositor. Ovipositor extrusion is a display that occurs exclusively in the context of courtship. A caveat of this work is the large number of neurons that are affected by this manipulation. Is the low copulation rate a result of an issue created by silencing large numbers of neurons? The capacity to recognize the male partner does not seem to be affected and the phenotypes we observed are revealed only in the context of courtship which strongly suggests that we specifically affected parts of the natural receptivity circuit.

How can changes in activity of *apterous* neurons affect female velocity during courtship? The best understood cue from the male that shapes female velocity is the song^57^. It is unlikely though that *apterous*-silenced females have impaired hearing. *ap*^*GAL4*^ labels very faintly the region that is innervated by first and second order auditory neurons, AMMC. Third order auditory neurons innervate the ventral lateral protocerebrum (VLP). We silenced the VLP neurons (and a few other neurons) that express *apterous* and did not observe a receptivity phenotype (Figure S3, intersection between *apterous* and glutamatergic neurons). Moreover, flies with impaired hearing do not copulate within the time of our analysis^25^. Most likely *apterous* neurons are involved in integrating sensory cues provided by the male that would lead to a receptive state of the female and over time result in a decrease of female velocity.

We have also uncovered a role for *apterous* neurons in controlling egg laying, another critical aspect of reproductive behaviour. Females that have mated and then have their *apterous* neurons in the brain inhibited lay very few eggs unlike control females. Classic gynandromorph studies pointed to an egg laying suppression focus in the brain^58^ but here we seem to have identified a focus that promotes egg laying when active. A recent study implicates the *doublesex*-positive and female-specific PMN2 descending neurons in oviposition behaviour^59^. *apterous* neurons are neither *doublesex* positive nor descending, so it is reasonable to assume that we identified a novel set of neurons.

In conclusion, our findings contribute to understanding female receptivity and highlight the central role of female speed modulation during courtship. It will be interesting to elucidate in the future the role of the different *apterous* clusters and reveal how they interact with the sexual specification circuits.

## Methods

### Fly stocks

Flies were raised in standard cornmeal-agar medium at 25 °C and 70% relative humidity in a 12 h:12 h dark:light cycle, unless otherwise indicated. Fly strains and sources are as follows: *ap*^*md544*^ 60; *UAS*-*Kir2*.1^40^; *tub-GAL80*^*TS*^ 41; *UAS-CD8-GFP*^61^; *20xUAS-CD8-GFP*^62^; *UAS-AUG-DsRed* (Kasuya & Iverson, Flybase); *Otd-nls:FLPo*^44^, provided by D. Anderson*; UAS* > *stop* > *CD8-GFP*^63^*; UAS* > *stop* > *Kir2*^20^, provided by Y.N. Jan*; fru*^*LexA*^ 52, provided by D. Mellert*; LexAop-CD2-GFP*^64^; *8xLexAop2-FLP*_*L*_^65^; *UAS-dcr-2*^66^; *UAS-tra-2*^*IR*^ 66; *elav-GAL80*^20^; *repo-GAL80*^67^, provided by T. Lee*; OK107-GAL4*^68^; *norpA*^36^,^43^; *tub* > *stop* > *GAL80*^45^; *actin5C-GAL4*^69^; *VGlut*^*MI04979*^*-LexA:QFAD, GAD1*^*MI09277*^*-LexA:QFAD, Cha*^*MI04508*^*-LexA:QFAD*^70^. *Wild-type: Canton-S (CS) and* Dickinson Lab (DL). Detailed genotypes can be found in the Supplementary Information.

### Neuronal silencing

For temporally restricted neuronal silencing experiments, female flies were raised at 18 °C for 6 to 12 days. Tester flies were subsequently incubated at 30 °C for 24 h, whereas control flies were maintained at 18 °C. Both control and tester flies were shifted to 25 °C, 24 h before the behavioural assay for acclimation and to prevent the effect of temperature treatment in the behaviour. Target males were wild-type CS flies aged for 3 to 8 days at 25 °C. Silencing experiments without temporal restriction were performed in female flies raised at 25 °C for 3 to 12 days.

### *NorpA* mutant receptivity

For experiments with *norpA* mutant females, flies were aged for 3 to 6 days at 25 °C. Wild-type DL flies were used as background control in these experiments. Target males were CS flies aged for 3 to 5 days at 25 °C.

### Masculinization of *apterous* neurons

‘Masculinization’ of *apterous* neurons was achieved by knocking down endogenous Tra-2, which is required for the establishment of female specific morphology and neural circuitry^55^. The efficiency of the short hairpin RNA was evaluated by knocking down Tra-2 in all cells, using an *actin5C-GAL4* driver. The females exhibited male-like external sexual morphology (data not shown). To improve the knockdown efficiency, the *UAS-dcr-2* transgene was co-expressed with the short hairpin RNA. Flies were raised at 25 °C for 4 to 8 days. Target males were CS flies aged for 4 to 8 days at 25 °C.

### Egg laying

Females in groups of 5 were aged at 18 °C for 6 to13 days before the assay. Females were mated by adding 3 males to each group during ~7 h. Virgin and mated tester flies were subsequently incubated at 30 °C for 24 h, whereas control flies were kept at 18 °C. Both control and tester flies were incubated overnight at 25 °C for acclimation, before being transferred to a plate containing apple medium. Groups of five females were kept in the plate at 25 °C, with 70% relative humidity, for 24 h before eggs were counted.

### Mating assays

In plexiglass chambers: Virgin females and males were collected soon after eclosion and kept isolated in individual vials until behavioural experiments. Single females were gently aspirated into circular plexiglass chambers (16 mm in diameter ×4 mm height) and subsequently paired with the male. Individual pairs were recorded for 30 min using SONY HDR-CX570E, HDRSR10E or HDR-XR520VE video cameras (1440 × 1080 pixels; 25 frames per second). A white LED backlight was used as light source (Edmund optics, cat# 83–875). A custom made software was developed to track the flies and automatically compute the time to copulation, whenever it occurred. All behavioural experiments were performed at 25 °C, with 70% relative humidity.

Small arenas: The plexiglass chambers were adapted to generate the small arenas. A mold with the dimensions 4 × 5 × 6 mm was inserted in the chamber that was then filled with 2% agarose. Removal of the mold generated the small arena. The experiments followed the pexiglass chamber protocol above. The position of the flies provided by the custom made tracking software was used to calculate velocity.

Conical shaped arena: We built an experimental setup consisting of a camera (Point Grey FL3-U3–32S2M-CS with a 5 mm fixed focal length lens (Edmund Optics)) mounted above a conical-shaped arena^47^. The conical arena is made of white Delrin with 11° sloped walls and 4 mm of height at the centre. Flies are able to walk in a circle of ~3 cm diameter. The arena is topped with a lid made of plexiglass. Movies were acquired in the dark, using an infrared 940 nm LED strip (SOLAROX) and a Hoya 49 mm R72 infrared filter. Flies were recorded in grayscale (1024 × 1024 pixels, 30 frames per second) for 20 minutes or until copulation occurred. All assays were performed at 25 °C with 70% relative humidity.

Virgin females were collected as soon as they ecloded, housed individually, and aged for 6–12 days at 18 °C. Temperature treatment was performed as stated above (see ‘Neuronal silencing’). Mated flies were treated as control virgin flies (kept at 18 °C). They were mated 24 h prior to the experiment, by incubating them with males for 1 hr. Immature virgin females were collected by aspiration and aged for 3 to 4 h at 25 °C before testing. Target males were naïve wild-type CS, aged for 4–8 days at 25 °C. For the experiments with paired females, target females were also wild-type CS, aged for 4–8 days at 25 °C.

We used the Caltech FlyTracker^71^ to track the positions, orientation and wing angle of both flies in each arena. The data were subsequently filtered to reduce jittering.

### Courtship index and percentage of copulation

In-house developed software was used to manually annotate the starting and ending time of each observed event of male courtship behaviour. We considered courtship behaviour moments when the male is either orienting toward the female, chasing the female, extending the wing, taping the female, licking the female or attempting copulation. The annotator was blind to the treatment of the video being annotated.

The courtship index is defined as the fraction of time the male spent courting the female from the moment he initiates the courtship and up to 10 minutes or until copulation. Percentage of copulation is the percentage of flies that mated within the duration of the video (either 20 or 30 min, see above).

### Ovipositor extrusion

In-house annotation software was used to annotate the moments when the female extrudes the ovipositor. The annotator was blind to the treatment of the video being annotated. Ovipositor extrusion is the time the female spent extruding the ovipositor over the total courtship duration.

### Quantification of locomotor activity

Female mean speed was calculated by averaging the speed within courtship and outside of courtship periods for each video. For the analysis we used the videos in which the courtship and non-courtship periods totalled at least 30 seconds in each one of them. Considering that the bulk of the female pausing bouts are less than one second long (Figure S9), 30 seconds provide a good sample of the female velocity. We eliminated the frames before initiation of courtship. To calculate the female mean speed during female-female interaction, we defined the female-female courtship-like periods based on the distance observed during male-female courtship. In male-female movies, when the flies are under 5.5 mm apart, in 95% of the frames they are courting (defined by manual annotation). When the flies are 5.5 mm apart or over, in 81% of the frames they are not courting. Based on this observation, we defined as courtship-like the frame where the two female are below 5.5 mm apart and non-courtship-like the remaining frames.

Pausing frames were defined as the frames in which the fly speed was less than 4 mm/s and the angular acceleration less than 15 rad/s^2^, as reported previously^10^. The fraction of time of female pausing corresponds to the number of pausing frames normalized to total courtship duration.

The speed before copulation was normalized by subtracting the mean speed of the 120–100 s period before copulation and dividing by the standard deviation within the final 100 s, as reported previously^11^. We grouped the normalized values into 30 frames bins and calculated the Spearman’s rank correlation (ρ).

### Wing extension index

We classified as wing extension wing angles above 60 degrees relative to the body axis. Wing extension index is the total number of frames where wing extension occurred over number of frames of courtship.

### Immunostaining and Microscopy

Tissues were dissected in phosphate-buffered saline (PBS), fixed in 4% paraformaldehyde in PBL (PBS and 0.12 M Lysine) for 30 min at room-temperature, washed 3 times for 5 min in PBT (PBS and 0.5% Triton X-100) and blocked for 20 min at room temperature in 10% normal goat serum in PBT (Sigma, cat# G9023). Subsequently, tissues were incubated with the primary antibodies in blocking solution, for 72 h at 4 °C. The following primary antibodies were used: rabbit anti-GFP (1:2000, Molecular Probes, cat# 11122); mouse anti-GFP 3E6 (1:500, Molecular Probes, cat# A11120); mouse anti-nc82 (1:10, Developmental Studies Hybridoma Bank); mouse anti-Dsx (1:100^72^); rabbit anti-DsRed (1:1000, Clontech, cat# 632496). Samples were washed 3 times for 5 min in PBT and incubated with either anti-mouse or anti-rabbit IgG conjugated to Alexa 594 or Alexa 488 for 72 h at 4 °C. To counterstain the female reproductive system, Alexa 594-conjugated phalloidin (Molecular Probes, cat# A12381) was used, diluted in blocking solution (1:50). Samples were washed 3 times for 5 min in PBT and mounted in VectaShield (Vector Labs, cat# H-1000). Confocal sections were acquired with Zeiss LSM 710 confocal microscope.

### Statistical analysis

Fisher’s exact test was performed to compare two different groups in the female receptivity assay. Egg laying, courtship index, ovipositor extrusion and female speed data was subjected to unpaired t test. The Mann Whitney test was used whenever the assumptions of the parametric test were not satisfied. Spearman’s rank correlation was used for the correlation of speed with time. Female receptivity and egg laying statistical analysis was performed with GraphPad Prism Software version 6.0 (GraphPad Software) and SciPy 0.18. The statistical analysis for the locomotor and annotated behaviours was performed with SciPy 0.18.

## Additional Information

**How to cite this article**: Aranha, M. M. *et al. apterous* brain neurons control receptivity to male courtship in *Drosophila melanogaster* females. *Sci. Rep.* **7**, 46242; doi: 10.1038/srep46242 (2017).

**Publisher's note:** Springer Nature remains neutral with regard to jurisdictional claims in published maps and institutional affiliations.

## Supplementary Material

Supplementary Information
