# Supplementary material for: apterous Brain Neurons Control Receptivity to Male Courtship in Drosophila Melanogaster Females
Source: Sci Rep. 2017 Apr 12;7:46242. doi: 10.1038/srep46242 (PMC5388873; doi:10.1038/srep46242)
Supplement: Supplementary Information [file srep46242-s1.pdf]

APTEROUS BRAIN NEURONS CONTROL RECEPTIVITY TO MALE COURTSHIP IN DROSOPHILA MELANOGASTER FEMALES

Márcia M. Aranha[2]*, Dennis Herrmann[1,2]*, Hugo Cachitas[2], Ricardo M. Neto-Silva[1,2], Sophie Dias[1,2], Maria Luísa Vasconcelos[1,2]

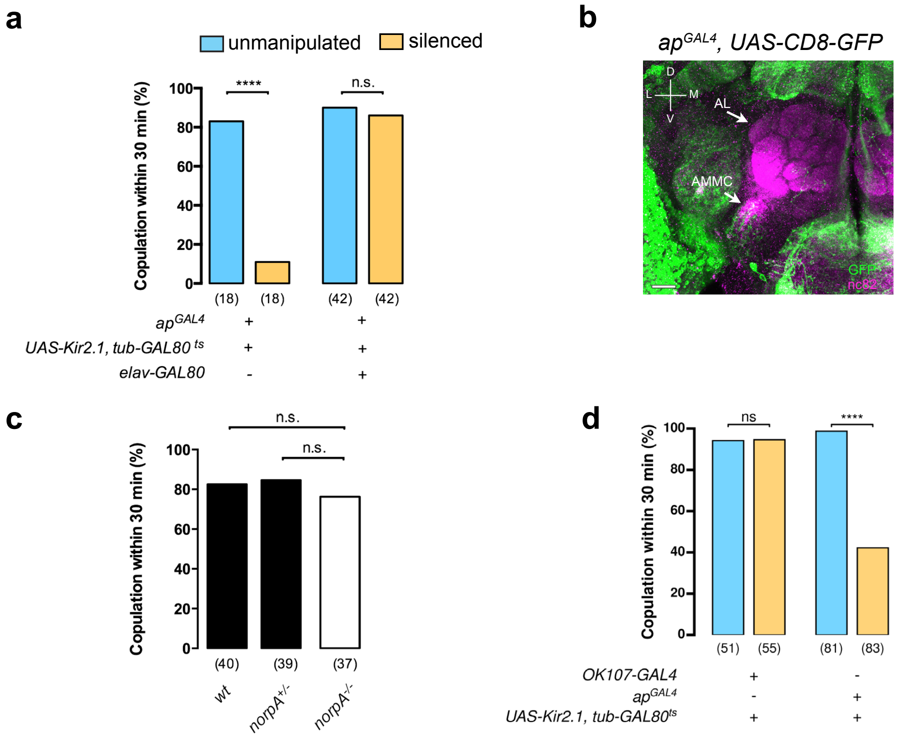

Figure S1. Reduced copulation from neuronal disruption of *apterous* neurons is unlikely to involve neurons related with vision or the mushroom body neurons.

(**a**) Mating of virgin females in the presence of an *elav-GAL80* transgene. Genotypes indicate females. n values shown in parentheses. n.s., not significant, ****$p<0.0001$, Fisher's exact test.

(**b**) Expression pattern of ap^{GAL4} in the female adult brain with a highlight in the antennal lobe (AL) and in the antennal mechanosensory and motor centre (AMMC). GAL4-driven expression is shown in green while the synaptic marker nc82 is shown in magenta. Scale bar represent 20 μm.

(**c**) Mating of blind females carrying a mutation in the *norpA* gene. Genotypes indicate females. n values shown in parentheses. n.s., not significant, Fisher's exact test.

(**d**) Mating of virgin females with *OK107-GAL4* neurons silenced. Genotypes indicate females. n values shown in parentheses. n.s., not significant, ****$p<0.0001$, Fisher's exact test

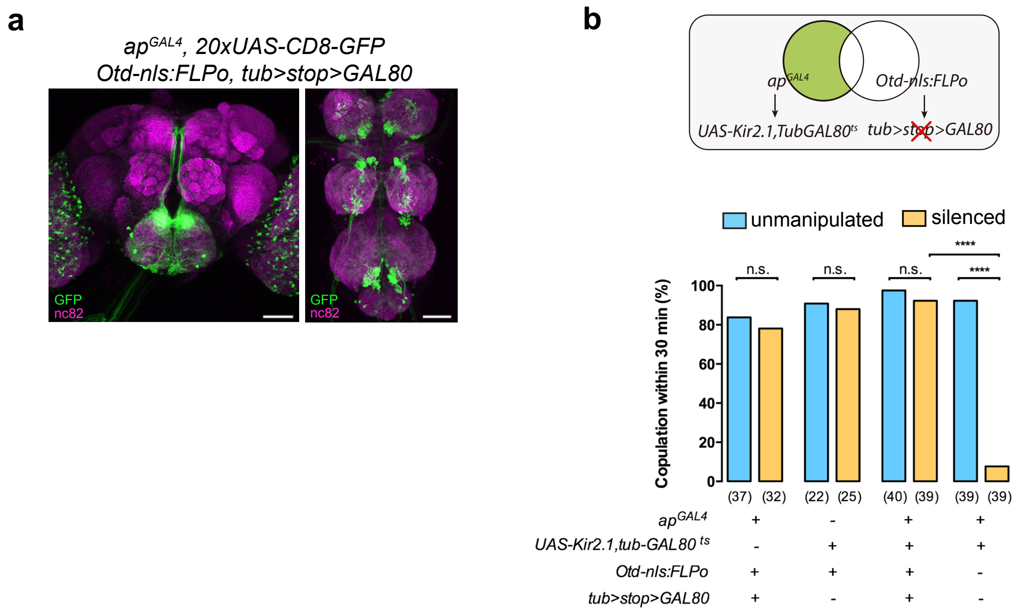

Figure S2. *apterous* neurons innervating in the subeosophageal zone and VNC are not involved in female receptivity.

(**a**) Expression pattern of *ap*^{GAL4}, excluding *Otd-nls:FLPo* intersecting neurons. GAL4-driven expression is shown in green while the synaptic marker nc82 is shown in magenta. Scale bar represents 50 μm.

(**b**) Mating of virgin females with a schematic representation of the manipulation employed depicted. Genotypes indicate females. n values shown in parentheses. n.s., not significant, ****$p<0.0001$, Fisher's exact test.

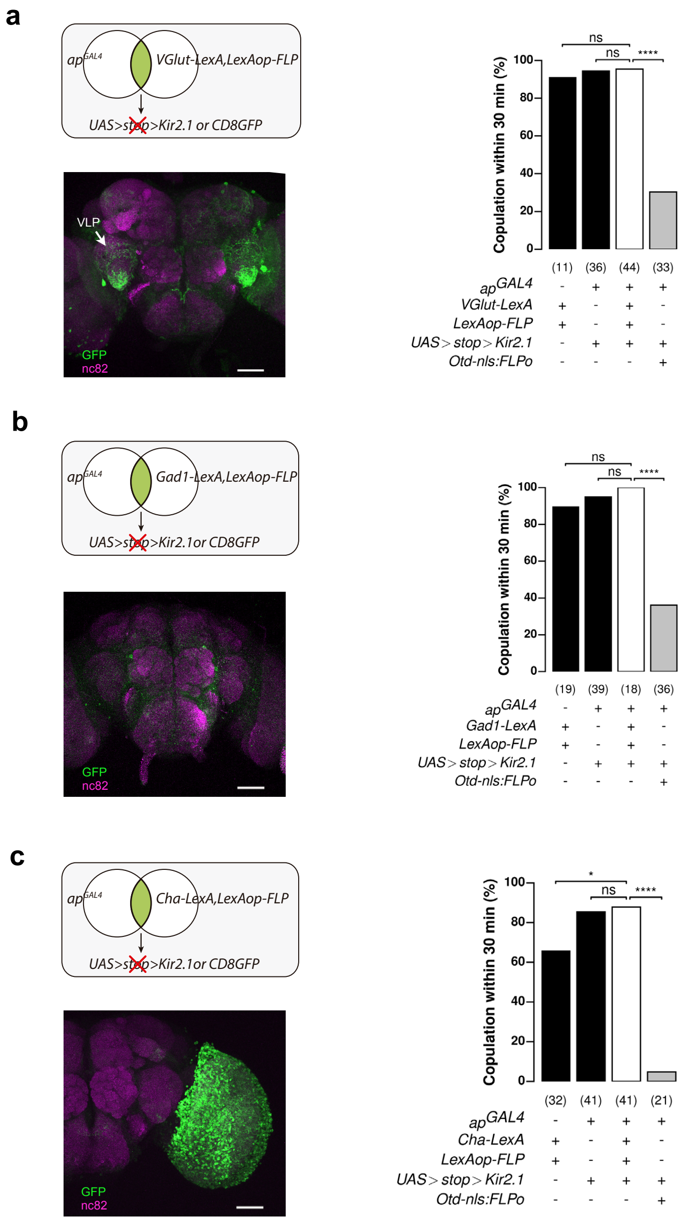

Figure S3. *apterous* neurons that intersect with cholinergic or GABAergic or glutamatergic neurons are not involved in female receptivity

Schematic representation of the manipulation employed, expression pattern and quantification of receptivity of virgin females in which the neurons at the intersections were silenced as follows.

(**a**) Neurons at the intersection of ap^{GAL4} and $VGlut^{MI04979}$*-LexA:QFAD*.

(**b**) Neurons at the intersection of $ap^{GAL4}$and $GAD1^{MI09277}$*-LexA:QFAD*.

(**c**) Neurons at the intersection of *ap*^{GAL4} and *Cha*^{MI04508}*-LexA:QFAD*. Intersecting neurons labelled in 3 to 4 day old females. Intersecting neurons were visualized with anti-GFP (green) and the tissue counterstained nc82 (magenta). Scale bar represents 50 μm. Genotypes shown correspond to those of virgin females. n values shown in parentheses. n.s., not significant, $*p<0.05$, $****p<0.0001$, Fisher's exact test.

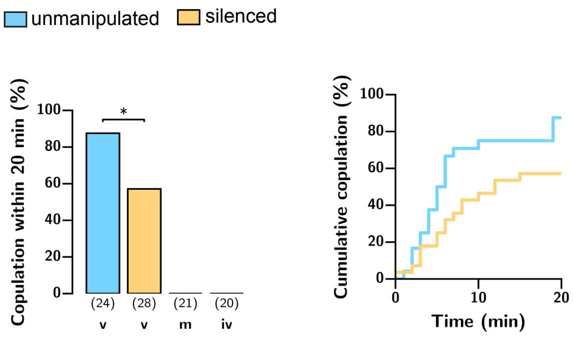

Figure S4. A similar reduction in mating of *apterous*-silenced females was observed when using conical-shaped arenas.

Mating of *apterous*-silenced female flies with the indicated mating status in conical-shaped arenas. v-virgin; m-mated; iv-immature virgin. n values shown in parentheses. n.s., not significant, *$p<0.05$, Fisher's exact test.

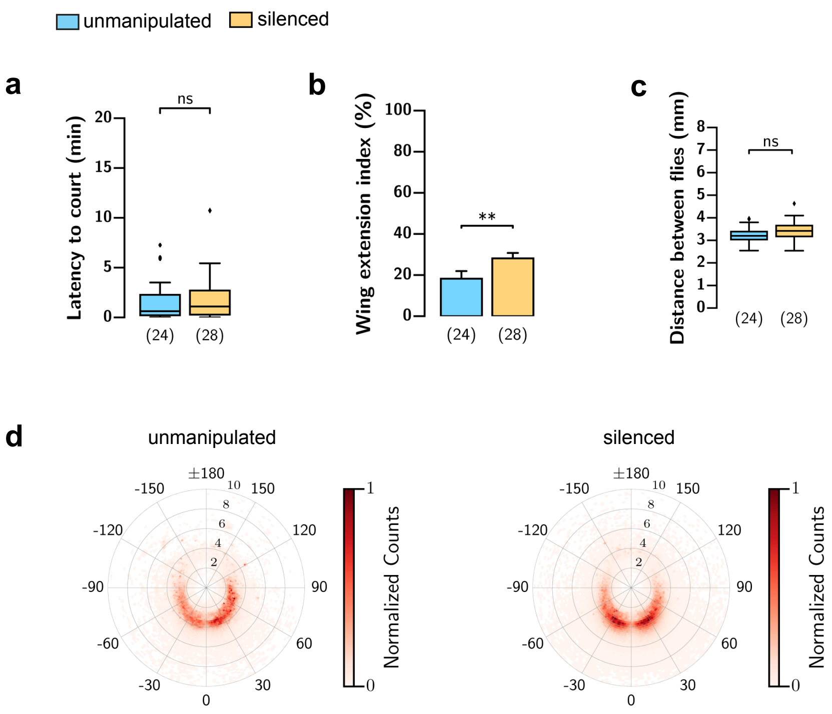

Figure S5. Male courtship parameters towards *apterous*-silenced virgin females are largely unaffected.

(**a**) Latency to court. n values shown in parentheses. n.s., not significant, Mann-Whitney U test.

(**b**) Wing extension index is a proxy for courtship song. n values shown in parentheses. $**p<0.01$, Mann-Whitney U test.

(**c**) Distance between the flies during courtship. n values shown in parentheses. n.s., not significant, unpaired t test.

(**d**)Male position relative to the female. Distance and angle of the male in each frame relative to the position of the female. Zero degrees represents frontal alignment of the male with the back end of the female. Distance between flies is shown in millimetres for each circle.

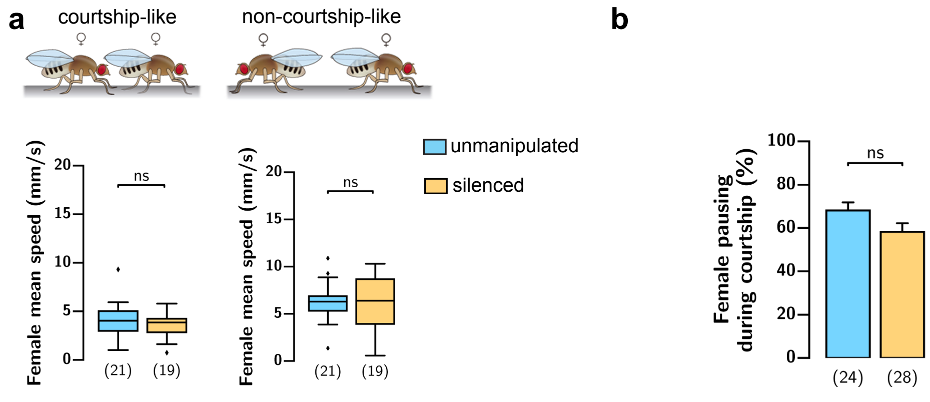

Figure S6. Additional locomotor features are not affected in *apterous*-silenced females

(**a**) Female velocity when in the presence of another female. Courtship-like moments were defined as the periods in which the distance between the female flies is below 5.5 mm. The periods in which the distance was equal or higher than 5.5 mm were classified as non-courtship-like periods. n values shown in parentheses. During courtship-like: n.s., not significant, Mann Whitney test. During non-courtship-like: n.s., not significant, unpaired t test with Welch's correction.

(**b**) Female pausing during courtship in male-female videos. n values shown in parentheses. n.s., not significant, unpaired t test.

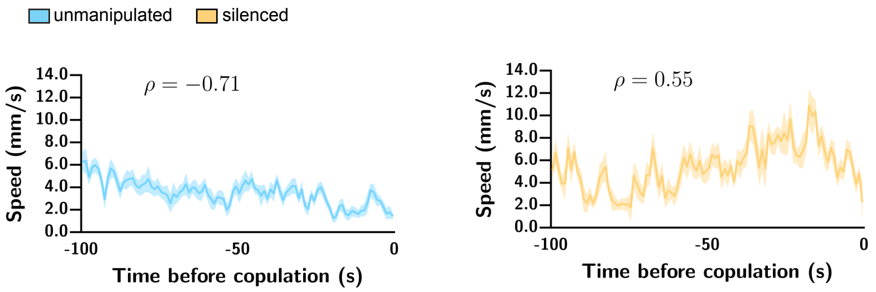

Figure S7. Progression of female velocity in 100s leading to copulation in control and *apterous*-silenced virgin females

To quantify the association between velocity and time to copulation we calculated rank correlations. Spearman's rank correlation: control females, 20 videos, frame bin, *p<0.0001*; *apterous*-silenced females that copulated, 15 videos, 30 frame bin, *p<0.0001*.

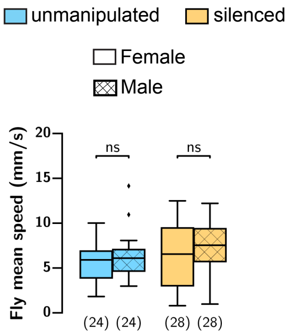

Figure S8. Female and male velocities in mating experiments are very similar

Comparison of male and female velocity in conical-shaped arenas during all experimental period or until copulation. n values shown in parentheses. n.s., not significant, Mann-Whitney U test.

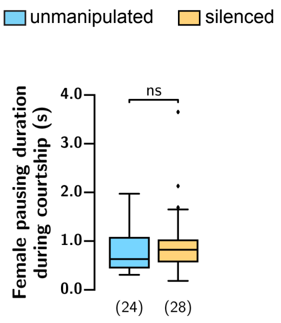

Figure S9. Female pausing duration

Duration of female pausing bouts during courtship in control and *apterous*-silenced virgins. n values shown in parentheses. n.s., not significant, unpaired t test.

Supplemental Experimental Procedures

Fly Stocks

Detailed genotypes of all strains used in the paper are as follows:

Figure 1.

<u>Panel b</u>
Females:
w^{1118}; ap^{GAL4}/+;+
w^{1118};+;UAS-Kir2.1, tub-$GAL80^{ts}$/+
w^{1118}; ap^{GAL4}/+;UAS-Kir2.1, tub-$GAL80^{ts}$/+

Males:
Canton-S wild-type

<u>Panel c</u>
Females:
w^{1118}; ap^{GAL4}/CyO;20xUAS-CD8-GFP/TM6b

Figure 2.

<u>Panel a</u>
Females:
Brain and VNC: w^{1118}; ap^{GAL4}/Otd-nls:FLPo;UAS>stop>CD8-GFP /+
Rep. system: w^{1118}; ap^{GAL4}/Otd-nls:FLPo;UAS>stop>CD8-GFP / tub-$GAL80^{ts}$

<u>Panel b</u>
Females:
UAS>stop>Kir2.1/ w^{1118}; ap^{GAL4}/+;+
w^{1118}; Otd-nls:FLPo/+; tub-$GAL80^{ts}$/+
UAS>stop>Kir2.1/w^{1118}; ap^{GAL4}/Otd-nls:FLPo; tub-$GAL80^{ts}$/+

Males:
Canton-S wild-type

Figure 3.

<u>Panel a</u>
Females:
UAS>stop>Kir2.1/ w^{1118}; ap^{GAL4}/+;+
w^{1118}; Otd-nls:FLPo/+; tub-$GAL80^{ts}$/+
UAS>stop>Kir2.1/w^{1118}; ap^{GAL4}/Otd-nls:FLPo; tub-$GAL80^{ts}$/+

<u>Panel c and d</u>
Females:
UAS>stop>Kir2.1/ w^{1118}; ap^{GAL4}/ Otd-nls:FLPo; tub-$GAL80^{ts}$/+

Males:
Canton-S wild-type

Figure 4.

Females:
UAS>stop>Kir2.1/ w^{1118}; ap^{GAL4}*/ Otd-nls:FLPo; tub-*$GAL80^{ts}$/+

Males:
Canton-S wild-type

Figure 5.

Panel a
Females:
w^{1118}; ap^{GAL4}*/CyO;20xUAS-CD8-GFP/TM6b*

Panel b
Females:
w^{1118}; ap^{GAL4}*/LexAop-CD2-GFP;*fru^{LexA}*/UAS-AUG-DsRed*

Panel c
Females:
w^{1118};ap^{GAL4}*/UAS>stop>CD8-GFP;*fru^{LexA}*,8xLexAop2FLP/8xLexAop2FLP*

Panel d
Females:
w^{1118};ap^{GAL4}/+;fru^{LexA}*,8xLexAop2FLP/+*
UAS>stop>Kir2.1/w^{1118}*;+;8xLexAop2FLP/+*
UAS>stop>Kir2.1/w^{1118};ap^{GAL4}/+;fru^{LexA}*,8xLexAop2FLP/8xLexAop2FLP*
UAS>stop>Kir2.1/w^{1118};ap^{GAL4}*,Otd-nls:FLPo/+;8xLexAop2FLP/8xLexAop2FLP*

Males:
Canton-S wild-type

Panel e
Females:
UAS-dcr-2/w^{1118}; ap^{GAL4}/+;+
w^{1118};*UAS-*tra^{IR}/+;+
UAS-dcr-2/w^{1118}; ap^{GAL4}*/UAS-*tra^{IR};+

Males:
Canton-S wild-type

Figure S1.

Panel a
Females:
w^{1118}; ap^{GAL4}*/+;UAS-Kir2.1, tub-*$GAL80^{ts}$/+

elav-GAL80/w^{1118}*;* ap^{GAL4}*/+;UAS-Kir2.1, tub-*$GAL80^{ts}$*/+*

Males:
Canton-S wild-type

<u>Panel b</u>
Females:
w^{1118}*;* ap^{GAL4}*/CyO;20xUAS-CD8-GFP/TM6b*

<u>Panel c</u>
Females:
Dickinson lab wild-type
norpA/+;+;+
norpA;+;+

Males:
Canton-S wild-type

<u>Panel d</u>
Females:
w^{1118}*;* ap^{GAL4}*/+;UAS-Kir2.1, tub-*$GAL80^{ts}$*/+*
w; +; UAS-Kir2.1, tub-$GAL80^{ts}$*/+; OK107-GAL4/+*

Males:
Canton-S wild-type

Figure S2.

<u>Panel a</u>
Females:
w^{1118}*;*ap^{GAL4}*,Otd-nls:FLPo /Otd-nls:FLPo;tub>stop>GAL80/20xUAS-CD8-GFP*

<u>Panel b</u>
Females:
w^{1118}*;*ap^{GAL4}*,Otd-nls:FLPo/+;tub>stop>GAL80/+*
w^{1118}*;Otd-nls:FLPo/+;UAS-Kir2.1,tub-*$GAL80^{ts}$*/+*
w^{1118}*;*ap^{GAL4}*,Otd-nls:FLPo/Otd-nls:FLPo;tub>stop>GAL80/UAS-Kir2.1,tub-*
$GAL80^{ts}$
w^{1118}*;* ap^{GAL4}*/+;UAS-Kir2.1, tub-*$GAL80^{ts}$*/+*

Males:
Canton-S wild-type

Figure S3.

<u>Panel a</u>
Females:
w^{1118}*;* ap^{GAL4}*/* $VGlut^{MI04979}$*-LexA:QFAD;8xLexAop2FLP/UAS>STOP>CD8GFP*
w^{1118} *;*$VGlut^{MI04979}$*-LexA:QFAD/+;8xLexAop2FLP/+*
UAS>stop>Kir2.1/ w^{1118}*;* ap^{GAL4}*/+*

UAS>stop>Kir2.1/ w^{1118}; ap^{GAL4}/ $VGlut^{MI04979}$-LexA:QFAD; 8xLexAop2FLP/+
UAS>stop>Kir2.1/ w^{1118}; ap^{GAL4}/ Otd-nls:FLPo

Males:
Canton-S wild-type

Panel b
Females:
w^{1118}; ap^{GAL4}/ 8xLexAop2FLP;$GAD1^{MI09277}$-LexA:QFAD/UAS>STOP>CD8GFP
w^{1118}; 8xLexAop2FLP/+;$GAD1^{MI09277}$-LexA:QFAD/+
UAS>stop>Kir2.1/ w^{1118}; ap^{GAL4}/+
UAS>stop>Kir2.1/ w^{1118}; ap^{GAL4}/ 8xLexAop2FLP; $GAD1^{MI09277}$-LexA:QFAD /+
UAS>stop>Kir2.1/ w^{1118}; ap^{GAL4}/ Otd-nls:FLPo

Males:
Canton-S wild-type

Panel c
Females:
w^{1118}; ap^{GAL4}/ 8xLexAop2FLP; $Cha^{MI04508}$-LexA:QFAD /UAS>STOP>CD8GFP
w^{1118};8xLexAop2FLP; $Cha^{MI04508}$-LexA:QFAD
UAS>stop>Kir2.1/ w^{1118}; ap^{GAL4}/+
UAS>stop>Kir2.1/ w^{1118}; ap^{GAL4}/ 8xLexAop2FLP; $Cha^{MI04508}$-LexA:QFAD /+
UAS>stop>Kir2.1/ w^{1118}; ap^{GAL4}/ Otd-nls:FLPo

Males:
Canton-S wild-type

Figure S4, S5, S7, S8, S9.

Females:
UAS>stop>Kir2.1/ w^{1118}; ap^{GAL4}/ Otd-nls:FLPo; tub-$GAL80^{ts}$/+

Males:
Canton-S wild-type

Figure S6.

Panel a
Tester females:
UAS>stop>Kir2.1/ w^{1118}; ap^{GAL4}/ Otd-nls:FLPo; tub-$GAL80^{ts}$/+

Target females:
Canton-S wild-type

Panel b
Females:
UAS>stop>Kir2.1/ w^{1118}; ap^{GAL4}/ Otd-nls:FLPo; tub-$GAL80^{ts}$/+

Males:
Canton-S wild-type
